# Thoracoscopic mesh implantation as a definitive treatment approach for peritoneal dialysis-associated hydrothorax

**DOI:** 10.1007/s13304-022-01356-9

**Published:** 2022-08-21

**Authors:** Attila Nemeth, Migdat Mustafi, Godehard Friedel, Michael Sayer, Nils Heyne, Christian Schlensak, Ferruh Artunc, Volker Steger

**Affiliations:** 1 Department of Thoracic and Cardiovascular Surgery, University Hospital Tübingen, Hoppe-Seyler-Str. 3, 72076 Tuebingen, Germany; 2 Department of Internal Medicine, Division of Endocrinology, Diabetology and Nephrology, University Hospital Tübingen, Tuebingen, Germany; 3 Institute of Diabetes Research and Metabolic Diseases (IDM), Helmholtz Center Munich at the University Tübingen, Tuebingen, Germany; 4 German Center for Diabetes Research (DZD), The University Tübingen, Tuebingen, Germany

**Keywords:** Peritoneal dialysis, Pleuroperitoneal leakage, Hydrothorax, VATS, Mesh, Diaphragm

## Abstract

Pleuroperitoneal leakage with the formation of hydrothorax is a rare complication of peritoneal dialysis, usually necessitating termination of peritoneal dialysis. We hypothesized that implantation of a polypropylene mesh on the diaphragm using video-assisted thoracoscopic surgery might induce permanent closure of pleuroperitoneal leakage. We report a case series of *n* = 12 peritoneal dialysis patients with pleuroperitoneal leakage and right-sided hydrothorax who underwent video-assisted thoracoscopy with mesh implantation from 2011 to 2020. Pleuroperitoneal leakage had been confirmed before surgery by intraperitoneal administration of toluidine blue, contrast-enhanced computer tomography or glucose determination from the pleural effusion. Median time from the start of peritoneal dialysis to manifestation of pleuroperitoneal leakage was 52 days. Video-assisted thoracoscopic surgery revealed multiple penetration points in the tendinous part of the diaphragm in all patients, which appeared as blebs. These were closed by covering the whole diaphragm with a polypropylene mesh. In all patients, peritoneal dialysis was paused for three months and bridged by hemodialysis. After restarting peritoneal dialysis and a median follow-up time of 1.9 years, none of the patients experienced a recurrence of pleuroperitoneal leakage. This case series demonstrates that pleuroperitoneal leakage in peritoneal dialysis patients can be permanently closed using thoracoscopic mesh implantation and allows peritoneal dialysis to be continued as renal replacement therapy.

**Graphical abstract:**

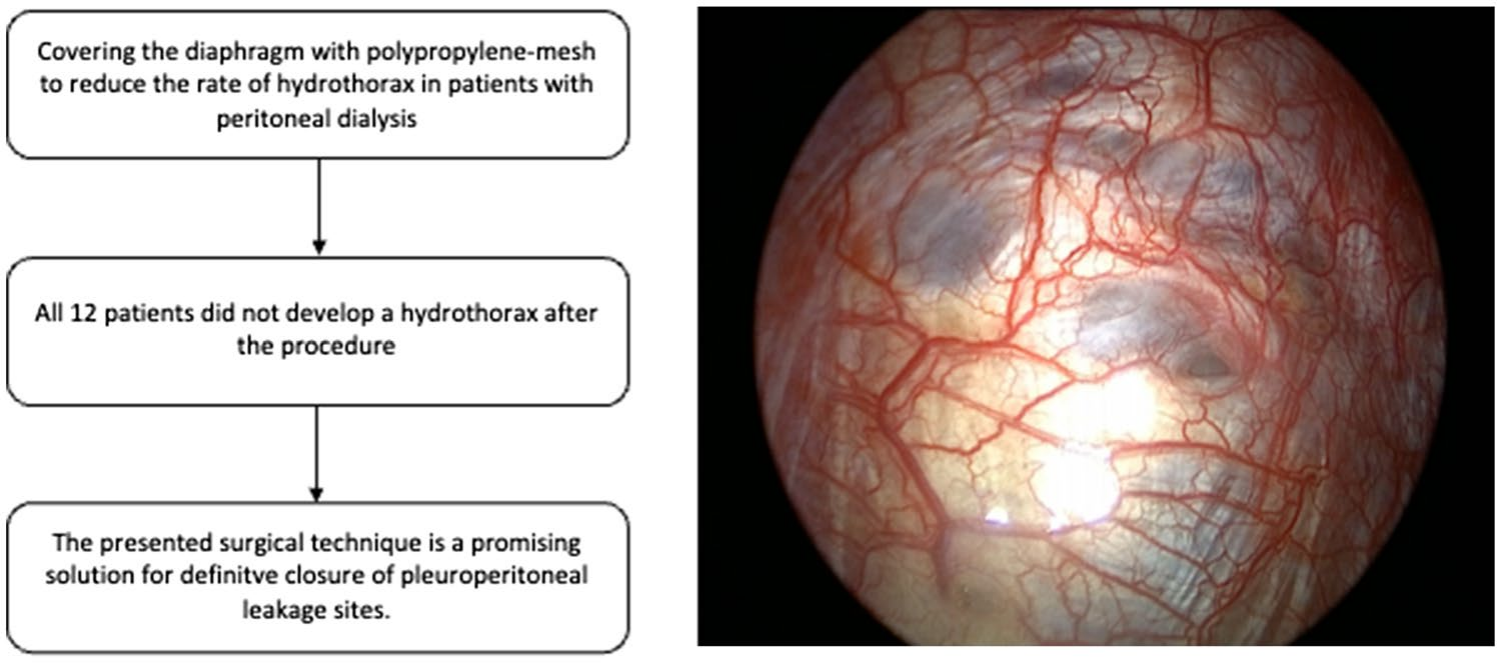

## Introduction

Leakage of the dialysate to adjacent regions and structures is a typical complication of peritoneal dialysis (PD) and most commonly manifests as swelling of the abdominal wall or inguinal hernia. Apart from sporadic and singular cases of dialysate leakage into the pericardium [1] or vagina [2], pleuroperitoneal leakage (PL) with the formation of hydrothorax is more common, as observed in 1.6–2.5% of large multicenter studies from Japan [3, 4]. Hydrothorax occurred within the first 2–4 weeks of PD in 40–50% of the cases, affecting the right side in 88% [3]. PL is thought to be caused by preformed weak points or defects of the diaphragm that eventually fail to withstand the increased intraabdominal pressure. A similar pathogenesis is assumed to form hepatic hydrothorax, occurring in up to 10% of patients with decompensated liver cirrhosis [5]. PL is confirmed by demonstrating the translocation of dialysate into the pleura by either computed tomography with a contrast agent–enhanced dialysate, scintigraphy with labeled albumin, or instillation of toluidine blue to stain the dialysate. In the absence of these methods, determining the glucose gradient, defined as the difference between the glucose concentration in the plasma and aspirate greater than 50 mg/dL, might be diagnostic as well [6].

Traditionally, PL was treated by pausing PD to allow spontaneous defect closure or with chemical pleurodesis [7]. However, these approaches have limited success by 50%. Recurrent pleuroperitoneal leakage necessitates the termination of PD and switch to hemodialysis (HD). Higher success rates have been reported using video-assisted thoracoscopic surgery (VATS) with either instillation of the pleurodesis agent [8] or closure of the diaphragmatic defects by direct suture [4]. Huang et al. first published a technique for treating hepatic hydrothorax by covering and enforcing the diaphragm using the parietal pleura with a non-resorbable polyethylene terephthalate mesh or the mesh alone [9]. After ten years of experience, the author reported a recurrence in 4 out of 47 patients (6.3%) after a median of 21 months [10].

We hypothesized that VATS-guided implantation of a polypropylene mesh instead of polyethylene terephthalate mesh on the diaphragm might induce permanent closure of PL, allowing the continuation of PD. We report on a case series of patients with PL who underwent VATS with our improved technique, implanting a polypropylene mesh.

## Patients and methods

### Study cohort

The retrospective study cohort consisted of 12 peritoneal dialyses (PD) patients with pleuroperitoneal leakage who underwent VATS with polypropylene (prolene^®^) mesh implantation at Tübingen University Hospital, Germany, between 2011 and 2020 (Table 1). 3 patients were treated at the nephrology division of the Tübingen University Hospital, and nine patients were referred from across Germany. All patients actively opted for surgery to continue PD as renal replacement therapy. After diagnosing PL, all patients were switched to HD for 3 months using a tunneled central venous catheter. After discharge, follow-up data were collected from patients and treating nephrologists by phone. The censoring date was May 1st, 2021.

**Table 1:** Patient characteristics and outcome

#	Sex	Age [y]	Renal disease	PD modality	Method of diagnosis	Time until diagnosis [d]	Hemithorax side	Outcome	Follow-up [y]
1	M	71	Glomerulonephritis	CAPD	Toluidin instillation	551	R	Continued PD until death from sudden cardiac death	1.9
2	F	61	Analgetics nephropathy	CAPD	Toluidin instillation	53	R	Continued PD until death	3.7
3	M	51	Glomerulonephritis	CAPD	Contrast-enhanced CT	47	R	Continued PD until death from sepsis	4.0
4	F	52	Hypertensive nephropathy	CAPD	Glucose gradient	25	R	Continued PD until switch to HD due to inadequate dialysis quality	4.4
5	M	70	Nephrosclerosis	CAPD	Toluidin instillation	43	R	Continued PD	4.1
6	M	65	Glomerulonephritis	CAPD	Toluidin instillation	212	R	Continued PD until switch to HD due to refractory overhydration	0.5
7	F	52	Polycystic kidney disease	CAPD	Glucose gradient	453	R	Continued PD	3.6
8	F	55	Polycystic kidney disease	CAPD	Contrast-enhanced CT	23	R	PD not reinitiated due to sigma diverticulitis	
9	M	63	Diabetic nephropathy	CAPD	Glucose gradient	51	R	Continued PD	1.8
10	F	59	Polycystic kidney disease	CAPD	Contrast-enhanced CT	90	R	Continued PD until death from liver failure	1.4
11	F	60	Focal segmental glomerulosclerosis	CAPD	Contrast-enhanced sonography	26	R	Continued PD	1.8
12	F	71	Polycystic kidney disease	CCPD	Contrast-enhanced CT	188	R	Continued PD until switch to HD due to peritonitis	0.8

The censoring date for read-out of the outcome was May 1st 2021

Abbreviations: *PD* peritoneal dialysis, *CAPD* continuous ambulatory PD, *CCPD* continuous cyclic PD, *CT* computer tomography, *HD* hemodialysis, *R* right side

### Surgical technique

The surgical procedure was performed under general anesthesia with double-lumen intubation, and patients were placed in the lateral decubitus position. Using a biportal video-assisted thoracoscopic approach (Fig. 2A), the lower port incision was created above the diaphragm in the midaxillary line and extended to a width of approximately 5 cm. Based on the preoperative confirmation of the diagnosis, no application of toluidine blue was necessary. Firstly, six to eight polypropylene sutures were placed on the margins of the diaphragm. Afterward, a polypropylene mesh was slidden down the sutures to cover the entire diaphragm. Through the lower port, a 24 Fr chest drain was inserted. Both ports were closed with a subcuticular (intracutaneous) suture. After the surgery, the patients were extubated in the operating room and transferred to the regular ward. Chest tubes were removed when the output fell below 200 mL per day.

## Results

**Fig. 1:**
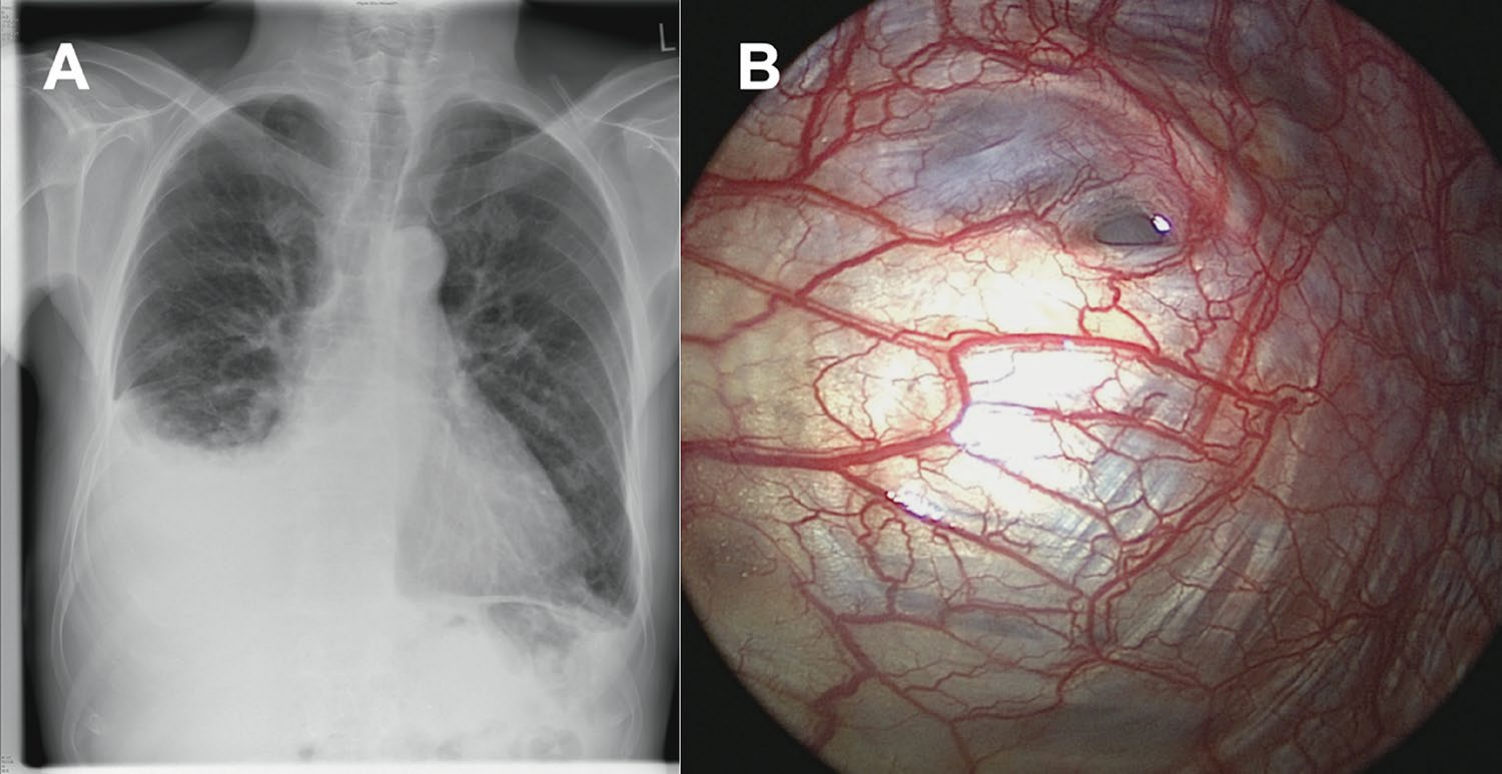
Right-sided pleuroperitoneal leakage in a 74-year-old male peritoneal dialysis patient. **A** Chest x-ray showing a right-sided pleural effusion. **B** Pleuroperitoneal leakage site demonstrated during video-assisted thoracoscopic surgery

The retrospective study cohort comprised 12 PD patients with PL receiving VATS at our University hospital (Table 1). The median age was 60 (range 51–71). 5 patients were male, and 7 were female. 11 patients were treated with continuous ambulatory PD (CAPD), and 1 patient was treated with continuous cyclic PD (CCPD). All patients had end-stage renal disease of various etiologies (*n* = 4 nephrosclerosis, *n* = 4 polycystic kidney disease, *n* = 3 glomerulonephritis, *n* = 1 diabetic nephropathy). All patients developed hydrothorax at the right side soon after initiation of PD (Fig. 1A), and the definitive diagnosis was made after a median of 52 days (range 23–551). Noteworthy, two patients developed hydrothorax in the second year on PD after 453 and 551 days, respectively. PL was diagnosed using CT or sonography using contrast agent–enhanced dialysate (*n* = 5) or dialysate stained with toluidine blue (*n* = 4) or determination of the glucose gradient (*n* = 3, Table 1).

**Fig. 2:**
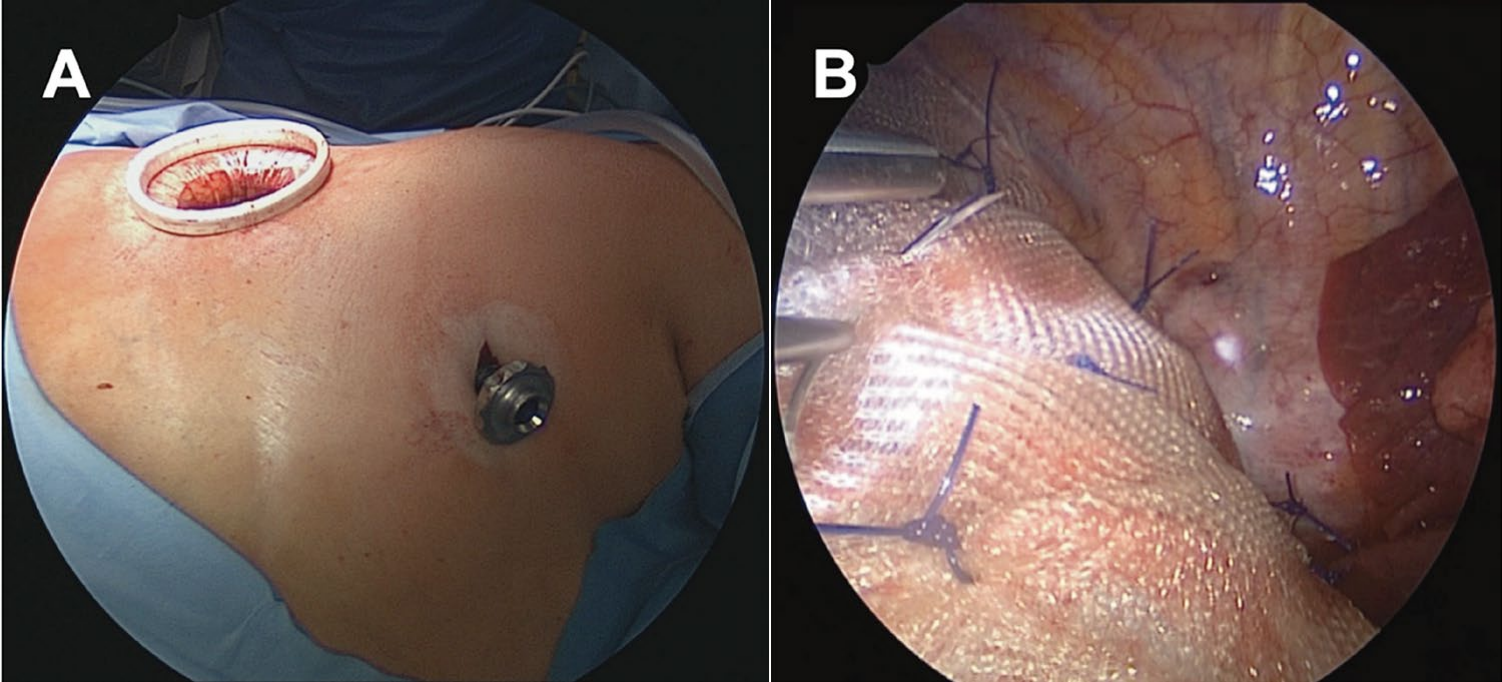
Thoracoscopic closure of pleuroperitoneal leakage site using mesh implantation. **A** Port placement for the biportal VATS technique. **B** Polypropylene mesh with sutures covering the diaphragm

After referral to the thoracic surgery department, all patients underwent VATS with a non-resorbable polypropylene mesh implantation (Fig. 2). The median surgery time was 97 min (67–120). Intraoperatively, most patients had macroscopic evidence of multiple defects in the diaphragm with a bleb-like appearance (Fig. 1B). There were no complications both intra- and postoperatively, and the patients were discharged after a median stay of 5 days [4–12].

**Fig. 3:**
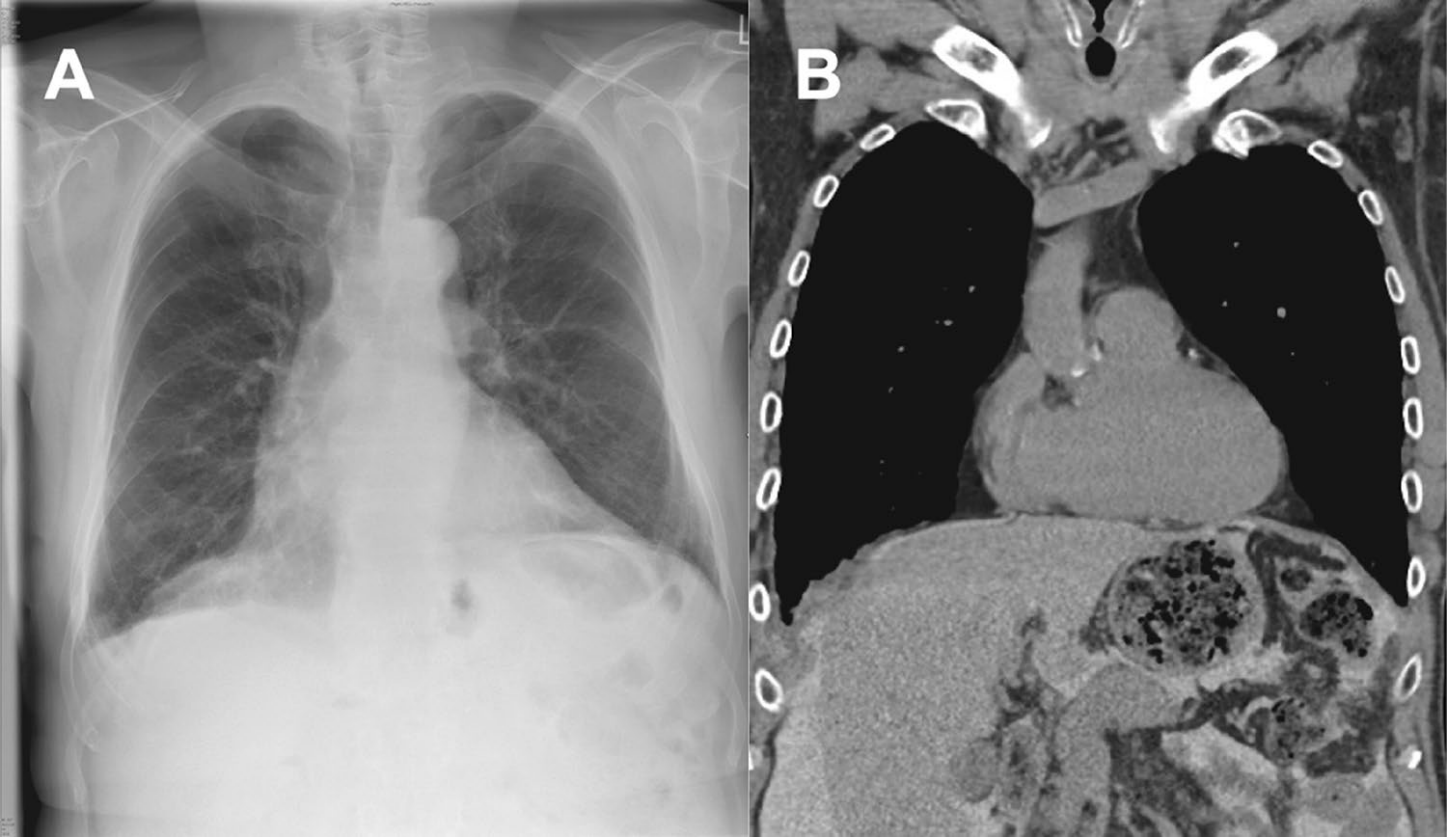
Radiologic appearance after mesh implantation in a 74-year old male peritoneal dialysis patient. **A** Chest x-ray 3 months after mesh implantation on the right diaphragm. **B** Computed tomography of the thorax 12 months after mesh implantation on the right diaphragm

PD could not be reinitiated in one patient due to perforating sigmoid diverticulitis. The remaining 11 patients reinitiated and continued PD without evidence of a recurrence of PL and hydrothorax during a median follow-up of 1.9 years (Table 1, Fig. 3). Two patients were still on PD at 4.1 and 4.4 years after surgery. 3 patients were switched to HD due to technical failure of PD (peritonitis, overhydration, inadequate dialysis quality) after a median of 2.2 years. 4 patients died from causes unrelated to PD or PL after a median of 2.7 years (Table 1).

## Discussion

This case series demonstrates the feasibility and efficacy of VATS-based polypropylene mesh implantation for repairing PL in PD patients. The surgery allowed all patients to resume PD after three months of HD. Continuation of PD as the preferred renal replacement therapy was the primary motivation for all PD patients with PL to undergo surgery for a nonvital indication. Patients benefitted from surgery as there was no recurrence of PL, and PD could be reinitiated in all patients. Compared with this study involving 12 patients, Matsuoka et al. reported the results of VATS-guided treatment in 11 PD patients with PL [4]. The authors used a stapler to close the lesions in the diaphragm and reinforced the diaphragm with an absorbable polyglycolic acid sheet. During follow-up, none of the surgically treated patients experienced recurrence, in contrast to eight of 14 PD patients treated nonsurgically. In another case series, 4 PD patients with PL were treated with VATS with ligation of the defect and covering the diaphragm using a polyglycolic acid sheet, over which adhesive chemicals were sprayed [11]. After a follow-up up to 46 months, there were no cases of recurrence. Further backed up by case reports [12–14], these results allow the conclusion that VATS-based treatment of PL appears superior to conservative therapy regarding prevention of recurrence and continuation of PD.

In most cases, PL occurred soon after the start of PD, suggesting preformed defects of the diaphragm [7]. In some patients in this study, we noticed multiple lesions intraoperatively that were even visible macroscopically. Smaller lesions can be visualized using toluidine-stained dialysate or intraperitoneal application of air [15]. Similar lesions have been described in the literature [4]. Huang et al. reported diaphragmatic blebs as the most common diaphragmatic defects in 46% of the patients with hepatic hydrothorax [10]. Most likely, the bleb-like appearance represents congenital weak points of the diaphragm that eventually cannot withstand the increased intraabdominal pressure caused by the dialysate.

This study reports a novel technique to implant a non-resorbable polypropylene mesh on a diaphragm. Considering the characteristics of available prosthetics, our choice was based on their strength, pore size, coating, and whether it is absorbable or not. Compared to abdominal or inguinal hernia repair, our ideal mesh should have low permeability and form adhesions on the parietal site [16]. Absorbable mesh offers flexibility but is dissolved over time and is therefore suitable only for a temporary closure of defects. The non-absorbable polypropylene mesh shows a higher and faster inflammatory reaction of the host tissue compared to the expanded polytetrafluoroethylene (ePTFE) mesh alone or the DualMesh, consisting of ePTFE and polypropylene [16]. Although DualMesh could preserve a better function of the pleura, polypropylene alone induces stronger lung adhesions with the whole diaphragm and therefore has a partial pleurodesis effect [17]. Furthermore, due to its smaller pore size, heavyweight polypropylene demonstrates an even stronger inflammatory reaction than lightweight prosthetics [16, 17].

As we did not attempt to suture the PL sites directly, visualizing the defects using intraoperative toluidine blue instillation was not required. Due to reducing the surgical steps to only cover the diaphragm with sutures on the margins, we successfully shortened the surgery time without compromising the results. Induction of adhesion minimizes the risk of future PL sites in other areas of the diaphragm and might be a superior approach to suturing single existing defects. However, compared to direct suturing, mesh implantation requires switching the patient to hemodialysis for three months to allow forming of the adhesion.

Additionally, the reduced number of sutures resulted in less postoperative pain and earlier mobilization of the patients. Therefore, hospitalization length was shorter than other reported techniques [4, 9, 10]. Huang et al. similarly used a mesh to treat patients with hepatic hydrothorax. To avoid creating suture holes, the mesh was used to cover the diaphragm without suturing [9, 10]. However, suturing prevents the dislocation of the mesh. Similar techniques of minimal-invasive mesh implantation have been described in women with catamenial pneumothorax [18–20]. Because of the possibility of endometrial nodules on the visceral pleura besides diaphragm fenestrations, additional pleurodesis to the mesh implantation is necessary. In patients with PL, due to PD, this is not needed. Although our experience with mesh implantation in women with catamenial pneumothorax is limited, adapting our technique along with a parietal pleurectomy could reduce the need for talc pleurodesis.

None of our patients had clinically evident reduced pulmonary function or diaphragm paralysis in the long term. However, we cannot rule out that the motility of the diaphragm might be slightly impaired after implantation of a mesh and induction of adhesion.

The single-center character limits our approach. However, it is conceivable that others might establish our approach since it is not dependent on custom-made equipment or items. Additionally, our experience is limited by the small sample size.

## Conclusion

In conclusion, we demonstrate that VATS-based diaphragmatic polypropylene mesh implantation is an effective treatment approach for PD-associated pleuroperitoneal leakage, allowing the continuation of PD without recurrence.
